# Can Genetic Pleiotropy Replicate Common Clinical Constellations of Cardiovascular Disease and Risk?

**DOI:** 10.1371/journal.pone.0046419

**Published:** 2012-09-28

**Authors:** Omri Gottesman, Esther Drill, Vaneet Lotay, Erwin Bottinger, Inga Peter

**Affiliations:** 1 The Charles Bronfman Institute for Personalized Medicine, Mount Sinai School of Medicine, New York, New York, United States of America; 2 Department of Biostatistics, Mailman School of Public Health, Columbia University, New York, New York, United States of America; 3 Department of Genetics & Genomic Sciences, Mount Sinai School of Medicine, New York, New York, United States of America; Medical University Hamburg, University Heart Center, Germany

## Abstract

The relationship between obesity, diabetes, hyperlipidemia, hypertension, kidney disease and cardiovascular disease (CVD) is established when looked at from a clinical, epidemiological or pathophysiological perspective. Yet, when viewed from a genetic perspective, there is comparatively little data synthesis that these conditions have an underlying relationship. We sought to investigate the overlap of genetic variants independently associated with each of these commonly co-existing conditions from the NHGRI genome-wide association study (GWAS) catalog, in an attempt to replicate the established notion of shared pathophysiology and risk. We used pathway-based analyses to detect subsets of pleiotropic genes involved in similar biological processes. We identified 107 eligible GWAS studies related to CVD and its established comorbidities and risk factors and assigned genes that correspond to the associated signals based on their position. We found 44 positional genes shared across at least two CVD-related phenotypes that independently recreated the established relationship between the six phenotypes, but only if studies representing non-European populations were included. Seven genes revealed pleiotropy across three or more phenotypes, mostly related to lipid transport and metabolism. Yet, many genes had no relationship to each other or to genes with established functional connection. Whilst we successfully reproduced established relationships between CVD risk factors using GWAS findings, interpretation of biological pathways involved in the observed pleiotropy was limited. Further studies linking genetic variation to gene expression, as well as describing novel biological pathways will be needed to take full advantage of GWAS results.

## Introduction

Cardiovascular events are frequently the final common endpoint of obesity, hypertension, hyperlipidemia, diabetes and kidney disease and modification of these traits remains the Standard of Care in the primary prevention of cardiovascular disease. The clinical management of Coronary Artery Disease (CAD), hyperlipidemia, hypertension, Type 2 Diabetes (T2D) and Chronic Kidney Disease (CKD) assumes an intrinsic interplay between these conditions and in particular, shared etiological and risk factors and is strengthened by their frequent co-existence in general patient populations. Looked at from an epidemiological perspective, the clinical picture is supported by extensive evidence. Both obesity and T2D have independently and in combination, been linked with increased risk of cardiovascular disease and death [1], [2], [3]. Diabetes increases the risk for cardiovascular disease 2-fold in men and 3-fold in women and outcomes following myocardial infarction are significantly worse in diabetic patients [4]. Diabetes is also the major risk factor for the development of chronic kidney disease and the leading cause of end-stage renal disease (ESRD) in the US [5]. Cardiovascular disease accounts for more than 50% of the mortality seen in ESRD patients [6]. Obesity has recently been implicated as an independent risk factor for the development of CKD, with one study estimating the risk of chronic renal failure may be up to 3 times higher in obese patients [7], [8]. The other significant risk factor for both cardiovascular and chronic kidney disease is hypertension. Thirty percent of American adults suffer from hypertension with less than half of those diagnosed having their blood pressure adequately controlled [9]. Uncontrolled and untreated hypertension is strongly associated with increased risk of cardiovascular mortality [10]. Blood lipid levels are significantly related to an individuals' risk of cardiovascular disease [11] and treatment with lipid-lowering medications, specifically HMG CoA reductase inhibitors (statins), is associated with decreased cardiovascular events in individuals at high and intermediate risk of cardiovascular disease [12]. It is also known that patients with hypertension tend to have a higher incidence of dyslipidemia, with higher triglyceride concentrations and lower high-density lipoprotein (HDL) concentrations than patients without hypertension [13]. Dyslipidemia has also been associated with all stages of chronic kidney disease [14]. CKD patients have characteristically elevated triglyceride levels, elevated LDL cholesterol levels, lower HDL cholesterol concentrations and elevated levels of lipoprotein(a) with a recent Cochrane systematic review suggesting that use of statins in CKD patients not requiring dialysis reduces all-cause mortality [15].

The apparent coexistence of these common conditions led to efforts to categorize these composite phenotypes, characterized by constellations of atherosclerotic risk factors, with obesity and hyperglycemia at their core. However there has been a lack of evidence to support the concept that these syndromes represent a distinct phenotype and that the risk conferred by a diagnosis of ‘Metabolic syndrome’ is any greater than the risk conferred by the sum of its' parts [16], [17]. The emergence of high-throughput genotyping technology and the hypothesis-generating genome wide association study (GWAS) have created an environment where disease-associated genomic information has been increasing at an unprecedented rate and provides an opportunity to assign biological reasoning to the established notion of shared risk.

Although large-scale GWAS have identified numerous significant SNP-trait associations, in the majority of cases the underlying pathophysiological mechanism has not been determined and in general, all known risk-variants combined explain only a small fraction of the observed heritability of these conditions [18]. To date, there has been limited success in identifying susceptibility loci for metabolic syndrome as an entity [19], however there have been numerous successes in identifying risk loci for CAD and it's clinically and epidemiologically-associated risk factors. This raises the possibility that some of these risk loci may be shared across these commonly occurring phenotypes and can account for their frequent coexistence.

Genetic pleiotropy refers to the phenomenon that single genes or variants may have an effect on multiple phenotypes [20]. Pleiotropy may occur directly as a shared consequence of the gene product or may be due to a signaling function affecting multiple downstream targets [21]. Previous studies have tested the idea of a shared genetic basis across multiple phenotypes in the context of GWAS findings. However, earlier assessments have been confined to the analysis of immune-mediated diseases [22], pancreatic cancer [23], hematologic and blood pressure traits [21], or unbiased screenings of a large number of human complex diseases and traits [20], [24].

**Figure 1:**
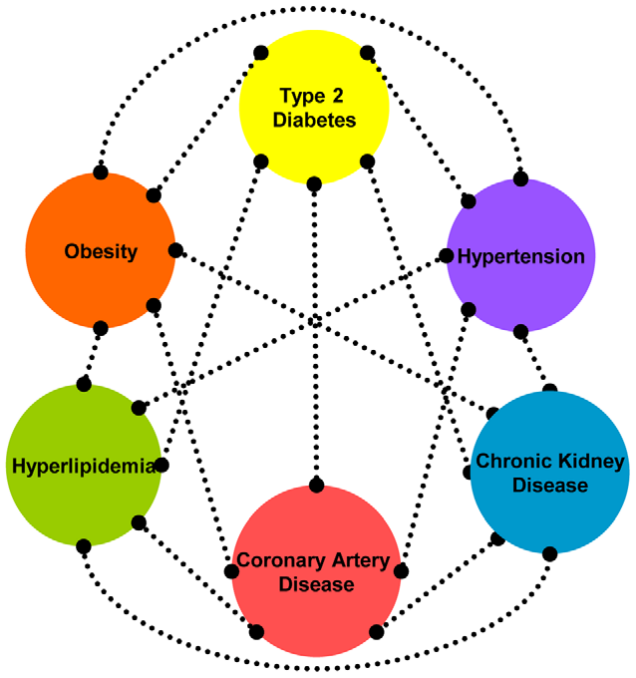
Bubble Chart representing the clinical and epidemiological relationship of cardiovascular risk factors. Connecting lines are unweighted (0/1) and indicate epidemiological relationships recreated from evidence presented in [1]–[15].

The relationship between obesity, diabetes, hyperlipidemia, hypertension, kidney disease and cardiovascular disease is established and indisputable when looked at from a clinical, epidemiological or pathophysiological perspective as illustrated in **Figure 1**. Yet, when viewed from a genetic perspective, there is comparatively little data synthesis that these conditions have an underlying relationship.

The goal of this study was to investigate the overlap of genetic variants that have been associated independently with each of these commonly co-existing conditions and intermediate risk factor phenotypes in an attempt to replicate the established notion of shared pathophysiology and risk through genetic pleiotropy. We conducted an analysis to evaluate the immediate interpretability of GWAS findings in this area of research using crude GWAS-derived genomic regions without processing or filtering results with regard to directionality of the reported associations or effect sizes.

## Methods

### Data Mining

We mined the online National Human Genome Research Institute's GWAS catalog [25], [26] for studies that conducted genome-wide screening for CAD, CKD, lipids, obesity and T2D and related traits using multiple search terms for each phenotype and traits related to that phenotype (last access June 10, 2011) (see **Table S1** for search terms).

The NHGRI GWAS Catalog is a curated resource for statistically significant SNP-trait associations (P<1×10^−5^) derived from GWAS publications. In order to control data quality, the catalog mandates standards for inclusion of published studies and is thus an excellent resource to study pleiotropy among GWAS-derived genetic variants [20].

We included meta-analyses of GWAS studies that reported candidate variants that had not been reported in the primary GWAS publications. Results of copy number variant analysis or studies that used family-based design in the discovery stage were excluded. Studies were also excluded because no SNPs were reported in the catalog. A closer review of these GWAS studies indicated that they had assayed less than 100,000 single nucleotide polymorphisms (SNPs) in the discovery stage or did not report SNP-trait associations with P-values of <1.0×10^−5^. In addition to the phenotype and associated SNPs, we extracted information on corresponding genes and race/ethnicity of the populations under study.

### Gene Annotation

We assigned genes to the associated SNPs by using the GWAS catalog's definition of *positional* genes that is based on the following criteria: a) if the SNP falls within a gene, that gene was assigned and b) if the SNP is intergenic, both the left-flanking and right-flanking genes were assigned irrespective of distance. In all instances where multiple SNPs were mapped to the same gene, only one gene per trait was chosen from each study. We also recorded author-reported genes.

We excluded human leukocyte antigen (HLA) loci that belong to the major histocompatibility complex (MHC) and contain a large number of genes related to immune system function in humans. The large extent of variability in HLA genes poses significant challenges in investigating the role of HLA genetic variations in diseases.

### Genetic Overlap

We identified all combinations of genes shared between 2 or more phenotypes using the assigned genes, for all studies combined and then for studies conducted in populations of European and African origin separately. To test for the robustness of the detected pleiotropy, ethnicity-pooled analyses were repeated using a SNP-trait associations that met a more stringent cutoff of P<1×10^−7^. Significance of the extent of pleiotropy was calculated with two methods. We first estimated the probability of genes associated with different phenotypes overlapping by chance alone by using the hypergeometric distribution with a pool of 4,105 genes, the number of non-HLA positional genes in the full GWAS catalog (as of June ′11), and positional gene lists of size as specified in **Table 1**. As described previously, these gene lists were derived from assigning the applicable reported SNPs to positional genes using the GWAS catalog definition. The hypergeometric approach assumes equal probability for each gene selected from the pool. Since the GWAS catalog contains multiple instances of genes, we then weighted the list according to how many times a gene appeared either because of unique SNPs mapped to that gene or unique phenotypes associated with that gene. Positional gene lists for each phenotype were randomly sampled 10,000 times from the weighted list of all 4,105 GWAS catalog genes and the number of gene intersections between phenotypes was used to calculate p-values.

**Table 1:** Number of studies, SNPs and genes by phenotype for all eligible studies.

Phenotype	N of Studies			N of SNPs			N of positional genes		
	All Ethnicities	European ancestry	African ancestry	All ethnicities	European ancestry	African ancestry	All ethnicities	European ancestry	African ancestry
BP and related traits	12	7	2	69*	50	10	85	61	16
CAD and related traits	15	13	1	90*	86*	3	101	93	6
T2D and related traits	33	23	1	145	110	2	137	106	3
Obesity and related traits	24	19	0	170	149	0	219	187	0
Lipids and related traits	24	18	1	176	135	31	141	92	44
CKD and related traits	11	6	3	75	41	32	105	54	49
Shared by 2+ phenotypes	9	5	1	16	16	0	44	36	2
Total	107	81	5	708	554	78	737	554	115

* includes a 4-SNP haplotype.

**Figure 2:**
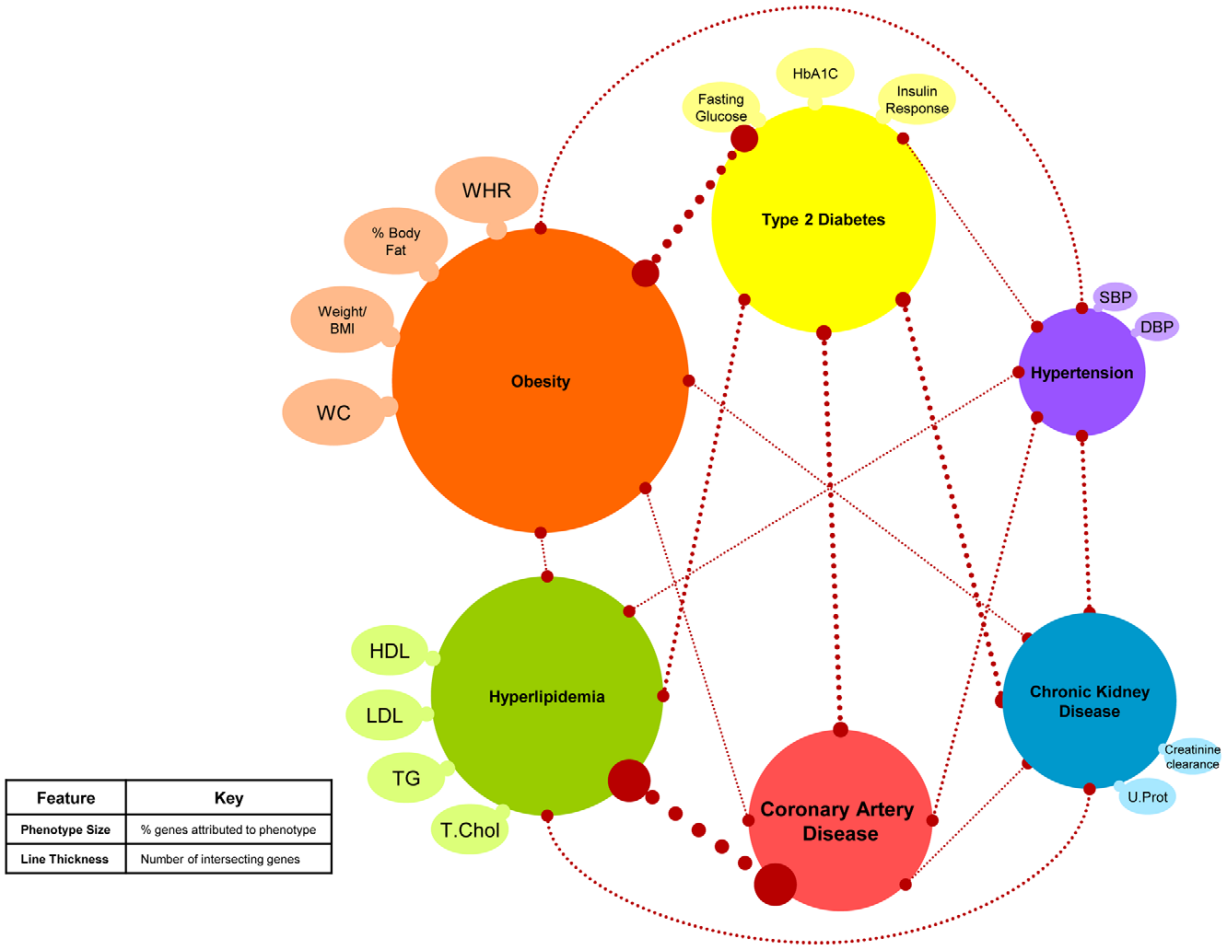
Bubble Chart representing the genetic relationship of cardiovascular risk factors including GWAS positional genes across all populations. The size of the phenotype is representative of the percentage of genes studied attributed to that phenotype. Line thickness is representative of the number of intersecting genes between two phenotypes.

We generated ‘bubble charts’ to visually represent the pairwise overlaps of genes associated with phenotypes (**Figure 2**, **Figures S1, S2, S3, S4**) and to have a comparison to the relationships presented in **Figure 1**. In these diagrams, the size of the phenotype bubble is representative of the percentage of genes studied attributed to that phenotype. Line thickness is representative of the number of intersecting genes between two phenotypes.

### Pathway Analysis

To identify common pathways shared between CVD-related phenotypes and CAD, Gene Relationships Among Implicated Loci (GRAIL) was used [27], [28]. GRAIL scores association signals by evaluating whether observed genomic regions are non-randomly linked to the other genes through word usage in PubMed abstracts, as well as the Gene Ontology and Gene Expression Atlas (Novartis) databases. GRAIL was chosen over other pathway-based genome-wide approaches (reviewed in [29]) for several reasons: 1) it seeks to infer relationships between genes, SNPs, or genomic regions without relying on predefined pathways or ontologies enabling to derive entirely novel networks of related genes; 2) it is superior to other analysis tools initially designed for microarray data that rely on large pathways and tend to have a greater chance of being statistically significant when GWAS data are considered [30]; 3) it analyzes regions defined by linkage disequilibrium (LD) and, therefore, relationships are only tested between genes in different regions minimizing any bias of LD between nearby genes representing the same association signal, and 4) it allows to visualize the resulting connections. We used the lists of pair-wise overlaps between all combinations of study phenotypes to assess the degree of connectivity between the implicated genes through word-similarity metrics [27]. To avoid publications that are influenced by disease regions discovered in the recent scans included in this study, we focused on PubMed abstracts published prior to December 2006, before the recent onslaught of GWAS papers identifying novel associations. However, in order to map all pleiotropic genes observed in our analyses, we repeated the analysis using abstracts published up to 2011, as well as the Gene Ontology (GO) and Gene Expression Atlas (Novartis) databases.

To accurately assess the statistical significance of the GRAIL connections, we conducted simulations in which we selected 100 sets of 38 positional genes (the subset of the 44 shared positional genes with most connections that were found in the GRAIL database) from both the unweighted and weighted list of all genes in the GWAS catalog (N = 4,105) that were also in the GRAIL database. Each of those 100 sets was scored with GRAIL to test for the likelihood of observing the same number or more significant connections at random.

## Results

Of the 125 potentially eligible articles in the GWAS catalog, we selected 107 that reported genetic associations with CAD and related risk phenotypes and met our inclusion criteria (**Table 1** **, Table S2**). We extracted a total of 708 associated SNPs, with 37 SNPs (5%) in coding regions, 311 SNPs (44%) in intergenic, 26 (4%) in near gene, 22 (3%) in UTR regions, and 312 SNPs (44%) in introns. These SNPs were assigned to 737 genes, with 101 genes associated with CAD and related traits, 137 with diabetes and related traits, 219 with obesity and related traits, 141 with lipids, 85 with blood pressure and related traits, and 105 with CKD and related traits (**Table 1**). Of the 107 eligible GWAS studies, 81 (76%) had initial scans performed in European and 5 (5%) in African-ancestry populations.

We detected 44 positional pleiotropic genes shared between at least 2 phenotypes (**Table 1**, **Table S3**). The largest number of genes was shared between CAD and lipids (14 positional genes). There were 9 genes shared between T2D and related traits and obesity-related traits. Using only positional genes, the extent of the CAD-lipid overlap reached statistical significance (P<0.001 by hypergeometric and 0.002 by weighted permutation test), whereas other 2-way overlaps did not (**Table S3**).

Seven positional genes showed pleiotropy across at least 3 CVD-related phenotypes in ethnicity-pooled analysis (**Table 2**). The most pleiotropy carried by a single gene was detected for *KLHL29* in the pooled analysis of all studies regardless of ethnic backgrounds and in stratified analyses that included only studies of individuals of African ancestry as defined in **Table S2**. Significant GWAS signals represented by *KLHL29* were found to overlap between blood pressure, lipids, CKD and CAD phenotypes, which is unlikely to occur by chance alone (P = 0.002 by hypergeometric and 0.050 by weighted permutation test). We also found additional suggestive 3-way overlaps of: 1) CAD, CKD-related traits, and lipids with *KLHL29* and *APOB* (P = 0.003 by hypergeometric and 0.082 by weighted permutation test) and 2) obesity, CKD-related traits, and T2D-related traits with *C6orf223* and *VEGFA* (P = 0.015 by hypergeometric and 0.251 by weighted permutation test). Three out of seven genes (*GCKR*, *C6orf223*, *VEGFA*) remained significant when only studies of European populations were considered.

**Table 2:** List of genes from all studies that showed three-way and four-way overlaps across CVD-related phenotypes.

Phenotype				GWAS Catalogue Genes	Hypergeometric (unweighted) p-value	Weighted p-value
BP	CAD	CKD	Lipids	*KLHL29*	**0.002**	0.050
BP	Lipids	Obesity		*ATG4C*	0.147	0.549
CAD	CKD	Lipids		*APOB, KLHL29*	**0.003**	0.082
CAD	Obesity	T2D		*DMRTA1*	0.165	0.601
Lipids	CKD	T2D		*GCKR*	0.110	0.458
Obesity	CKD	T2D		*C6orf223,VEGFA*	**0.015**	0.251

BP, blood pressure; CAD, coronary artery disease, T2D, type 2 diabetes.

Using pair-wise phenotype overlaps, we attempted to recreate all connections from the bubble chart, **Figure 1**, with information entirely from the GWAS catalog. We could replicate every relationship (line) using pleiotropy detected in the ethnicity-pooled analysis of positional GWAS genes (**Figure 2**). In studies of European populations only, positional genes did not independently replicate all connections (**Figure S1**). When exclusively African-ancestry studies were considered, we found that only 3 connections, between the blood pressure, lipids and chronic kidney disease phenotypes, were reproduced by one genomic region (*APOB*/*KLHL29*) (**Figure S2**).

Next, we limited the list of positional genes to reflect SNP-trait associations detected at a more stringent P<1×10^−7^ and found that, as expected, many overlaps disappeared, especially those with the blood pressure phenotypes, or decreased in number. Importantly, the overlaps that did not change were between the chronic kidney disease phenotypes and CAD, lipids and obesity, in addition to type 2 diabetes and lipids (**Table S3, Figure S3**). It is worth noting that less pleiotropic connections were also detected if only author-reported genes were used compared to positional genes (**Figure S4**).

To identify the key pathways suggested by the GWAS signals, we used GRAIL on positional pleiotropic genetic regions shared across at least two phenotypes. Based on abstracts published up to 2006, six out of 44 regions were dropped from the analysis as they were not found in the GRAIL database either due to the lack of sufficient literature or inconsistent mapping to Human Genomes (hg) 18. The lost genes were *CDKN2BAS*, the most replicated CAD locus (chromosome 9p21), *C6orf223*, *RPL12P33*, *EIF3FP3*, *UBA52P6* and *KLHL29* that showed the greatest level of pleiotropy in this study. We re-ran GRAIL using alternative data sources, such as GO and Gene Expression Atlas, and found matches for the 2 target genes (*KLHL29* and *CDKN2B*, an alias for *CDKN2BAS*). Nevertheless, no connections were established for these genes, whereas many other connections previously obtained through the literature search were lost. We screened interaction databases [31] and found no additional information on *KLHL29* and *CDKN2BAS*, supporting the notion that our knowledge of biological pathways is far from complete.

**Figure 3:**
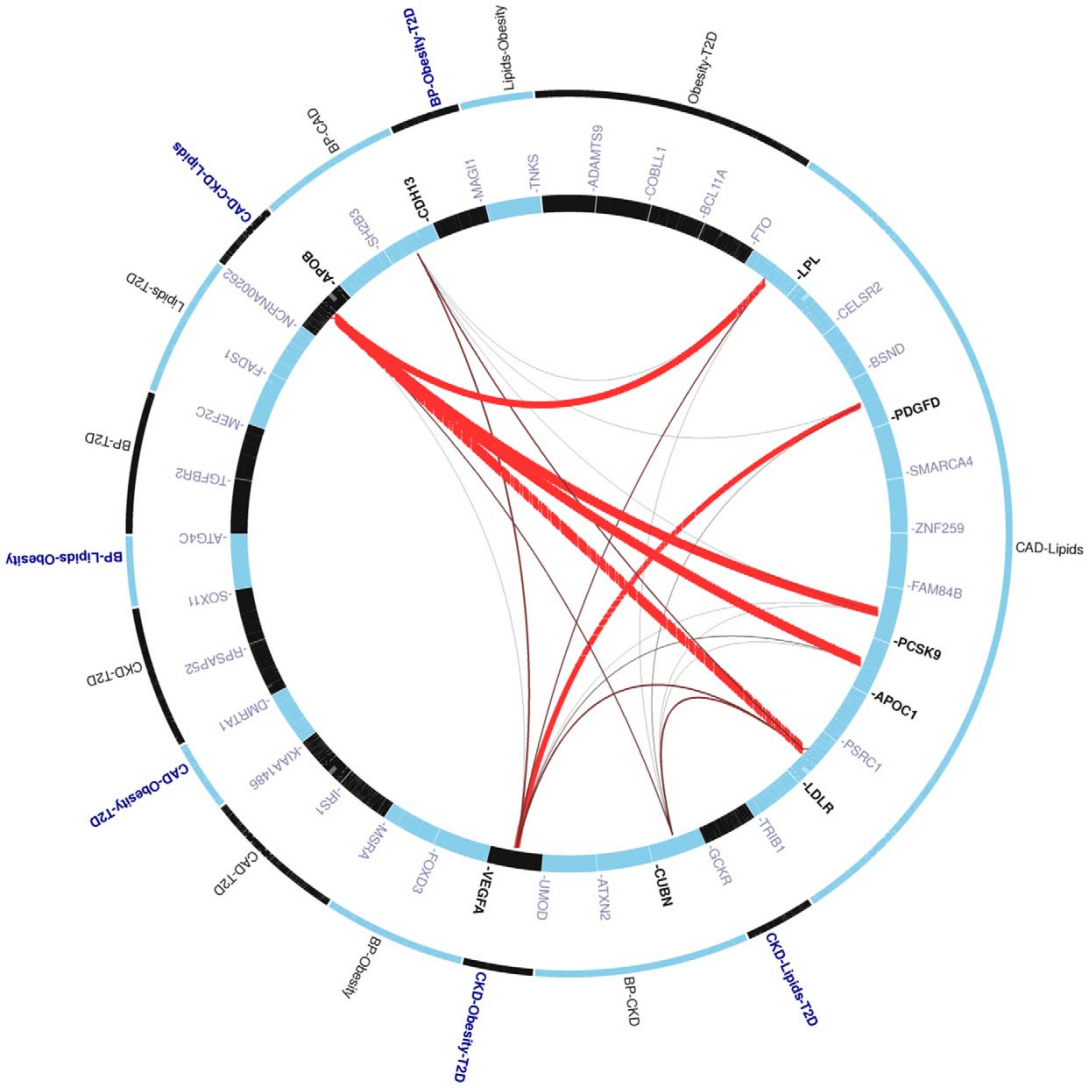
A plot of 38 positional genes that overlapped between at least two CVD-related phenotypes. Plotted using VIZ-GRAIL [28]. For each plot, phenotypic overlap is arranged along the outer circle; bold indicates three-way or four-way overlaps. Inner circle represents individual genes. The redness and thickness of lines connecting pairs of genes represent the strength of the connections with the thickness of the lines being inversely proportional to the probability that a literature-based connection would be seen by chance. Pathway-related links between 9 of 38 genes scored *P*<0.05 using GRAIL. To accurately assess the statistical significance of this set of connections, we conducted simulations in which we selected 100 sets of 38 genes and scored them with GRAIL. We determined that the likelihood of observing 9 hits with P<0.05 by chance is about 7%.

**Figure 3** demonstrates the most connectivity between the 38 positional genes by their enrichment in overlapping pathways that predominantly relate to lipid transport and metabolism. Nine of these genes were significantly scored with GRAIL indicating that they were non-randomly linked to the other genes through word usage in 2006 PubMed abstracts at P<0.05. In 100 simulated lists of 38 genes from the weighted GWAS catalog, the probability of observing 9 hits with P<0.05 by chance was 7%.

The 5 of 7 genes that showed pleiotropy across three or four phenotypes and were mapped by GRAIL did not reveal any inter-connectivity or connection to genes with an established pathway affiliation. When the analysis was repeated with abstracts published up to 2011 and inevitably informed by studies included in this analysis, we found that many of the 3+-way overlaps remained missing despite a dramatic increase in the amount of new information as expressed by the density of connections overall (**Figure S5**).

## Discussion

This year marks the fifth anniversary of the first large, well-designed GWAS that employed a dense SNP chip with varying opinions on the contribution of this study design to a better understanding of common disease susceptibility [32], [33], [34]. Despite the fact that over 2,000 loci have been found to be robustly associated with one or more complex traits, GWAS studies did not account for much of the individual trait's heritability. They generated reproducible hits often far from known genes that limited immediate translation of GWAS results into mechanistic understanding of phenotypic variation. Using genetic variants detected by GWAS studies of CAD and related traits, we sought to test whether unprocessed GWAS findings would support genetic pleiotropy that exists across several commonly co-existing morbidities and the extent to which the clinical and epidemiological notion of shared etiology can be reproduced. We report 44 genes identified by GWAS as being directly implicated in phenotypic variation, located upstream or downstream of the associated SNPs that were shared across at least two CVD-related traits. These overlapping genes recreated the established pathophysiological relationship between obesity, diabetes, hyperlipidemia, hypertension, kidney disease and CAD (**Figures 1** and **2**). However, this was only true if studies representing non-European cohorts were also included.

Previous reports have mined the GWAS catalog, testing the idea of a shared genetic basis across multiple phenotypes, such as pancreatic cancer, immune-mediated diseases, and hematologic traits, in the context of GWAS findings [20], [21], [22], [23], [24]. Instead, we conducted analyses focused on a cardiovascular disease domain while including intermediate risk factor phenotypes and co-morbidities to evaluate the immediate interpretability of unprocessed GWAS results in this area of research.

We found that genes within or flanking the reported SNPs independently replicated the clinical, epidemiological and pathophysiological notion of cardiovascular risks in the ethnicity-pooled dataset. However, when only studies of European populations were included, some of the relationships were lost (**Figure S1**). The fact that only 24% of the eligible studies were conducted in non-Europeans emphasizes the value of genetic studies in diverse ethnic/racial groups. When only studies of African ancestry cohorts were included in the analyses, we found that only 3 connections, between the blood pressure, lipids and chronic kidney disease phenotypes, were reproduced (**Figure S2**).

Several genes showed an overlap between at least three CVD-related traits (**Table 2**). Notably, one region on chromosome 2p24.1, in the proximity of two genes, Apolipoprotein (*APOB*) and kelch-like protein 29 (*KLHL29*), revealed the most extensive pleiotropy across multiple phenotypes, such as blood pressure, lipids, CKD and CAD. While *APOB* is well known for coding the main apolipoprotein of chylomicrons and low density lipoproteins, *KLHL29*'s function is widely unknown. Consistent signals pointing to that genomic area included a *KLHL29* intronic SNP and several intergenic SNPs positioned 19–2,323 bp from either gene, both imputed and typed, suggesting that both genes can be involved. Notably, *KLHL29* was only mapped to this genomic region in studies of African ancestry. This observation could suggest ethnic/racial differences in LD in the region or an ethnic-specific variant associated with multiple risks. Recently, there have been reports of variants associated with risk in specific ancestral populations, particularly in African-ancestry populations for diabetes [35], [36], kidney disease [37], and hypertension [38]. These discoveries could explain in part the increased prevalence of certain diseases in particular ancestral populations. However, whilst racial disparities or population differences in disease prevalence may correlate with differences in allele frequency resulting in different association signals, we were unable to effectively control for differences in allele frequency as the unit of analysis in our study was gene rather than SNP. Additionally, as the majority of GWAS-derived SNPs are not causal and population differences may be attributed to differences in LD structure, a lack of association in non-European populations may demonstrate ascertainment bias of GWAS markers rather than true population differences. Targeted re-sequencing should be conducted in multiple populations in an attempt to identify potential functional variants that generated the observed association signals.

We used pathway-based analysis as implemented in GRAIL to identify subsets of positional pleiotropic genes, shared across at least 2 phenotypes, involved in similar biological processes. GRAIL uses abstracts from the entirety of the published scientific literature to look for relatedness among genes within disease regions that may represent key pathways. We undertook this approach to capture both clearly established close gene relationships and potentially undocumented or distant ones. We found that the strongest connections were between genes involved in lipid transport and metabolism, such as *PCSK9*, *LDLR*, *LPL*, *APOB*, *APOC1* (**Figure 3**), significantly contributing to the GRAIL results. Among other significant connections was a link between the two growth factors, *PDGFD* and *VEGFA*, that belong to the platelet-derived growth factor/vascular endothelial growth factor (PDGF/VEGF) family implicated in a variety of functions in vertebrates, especially angiogenesis. Defects in *VEGFA* have been shown to be associated with diabetic retinopathy, diabetic nephropathy leading to end-stage renal disease and diabetic neuropathy. These genes were also connected to the lipid genes by GRAIL.

It is of interest that many pleiotropic GWAS loci had no relationship to each other or to genes with established functional connection regardless of how current the reference data. An incomplete gene function annotation and limited knowledge of biological pathways could potentially explain this finding. Despite considerable advances in expression quantitative trait loci (eQTL) research, there are questions about the completeness of the eQTL databases. Most of the human eQTL studies to date have analyzed cell types in blood, because these are the most readily available tissues, only recently moving to a wider variety of tissues such as cortical, adipose and liver tissues [39], [40], [41]. This reality prevented us from formally evaluating the contribution of eQTLs to genetic pleiotropy. Further studies will help elucidate pathways whose relevance to a particular disease or trait was previously unsuspected.

We sought to analyze the data in as unbiased a way as possible. To this effect, we did not include metabolic syndrome as a CVD-related phenotype, as its definition encompasses two or more of the included phenotypes and would thus positively bias any overlapping gene lists. We also excluded catalog genes that were reported by investigators who may have had pre-existing notions about disease causality, and relied only on positional information provided by the catalog. Of note, the complete replication of the established relationships was not reproduced when the analysis was limited to associations that reached genome-wide significance (P<1×10^−7^) or based on author-reported genes instead of positional genes.

This study has several limitations. We mined data exclusively from the NHGRI GWAS catalog, which includes data on published GWAS studies meeting pre-specified criteria. The catalog does not include variants derived from candidate gene or linkage studies and as such, variants discovered through these means that may exhibit pleiotropy were not included in our analysis. Similarly, we could only assess pleiotropy in the context of which phenotypes have been already studied, thus the absence of pleiotropy may denote insufficient data rather than true absence [42]. Conversely, it is possible that the degree of pleiotropic findings are artifactual because the implicated diseases have been explored in greater depth [20]. Furthermore, we could not control for gene size, which may affect the likelihood of observing statistically significant associations, as this inherited bias is present from the ascertainment of markers on GWAS arrays through to the reporting of association results in the GWAS catalog. Nevertheless, we limited adding to this bias by only including one instance of any gene that could be represented by multiple SNPs per phenotype in the analysis. Additionally, it is rare for causal variants to be identified by GWAS and, in many cases; variants in LD with the true causal variant are recorded in the catalog. These may in turn have been mapped to alternate genes in our analysis and may have affected the observed pleiotropy. It is possible that we included GWAS studies that used the same samples to study different phenotypes. Also, consistent with other studies examining pleiotropy in the GWAS catalog [21], we did not address the directionality of the reported associations, nor did we consider the level of statistical significance (other than the sub-analysis at the more stringent threshold) or their effect sizes. The goal of this study was to determine if it is possible to replicate an indisputable notion of commonly co-occurring CVD-related conditions using crude GWAS-derived genomic regions. Future studies will be needed to determine whether these genetic risks act independently, in synchrony or whether antagonistic pleiotropy exists between these phenotypes. The choice of the genotyping platform could have biased our results. Nevertheless, the top pleiotropic region, *APOB*-*KLHL29*, has been detected through the imputed and typed SNPs available from both Affymetrix and Illumina genotyping platforms. Furthermore, although variability in phenotypic characterization of CAD and related traits used by various GWAS studies may have affected our results, it has been shown that differences in phenotype definition in CAD have a small effect in between-study heterogeneity [43]. Another challenge of our study was that genes clearly implicated in the pleiotropy were not fully annotated with respect to function. That is, *KLHL29*, a gene within our most substantive pleiotropic region, as well as 5 other pleiotropic genes including the most widely replicated CAD locus on 9p21, were not found in the GRAIL databases and therefore, we could not examine whether their function is connected to that of other pleiotropic genes. For these genes, greater efforts will be required to chart new paths that could eventually lead to the most novel and important insights.

## Conclusions

Whilst we recreated the established pathophysiological relationship between obesity, diabetes, hyperlipidemia, hypertension, kidney disease and cardiovascular disease using genetic regions detected by GWAS, many of the observed pleiotropic genes could not be linked to each other or to known biological pathways. Further studies are needed to expand gene expression databases, characterize new pathways and improve gene annotation in order to take full advantage of GWAS findings.

## Supporting Information

Table S1 Search terms used to select studies from the GWAS catalog by major phenotype.
(DOCX)

Table S2 List of included studies.
(XLSX)

Table S3 List of GWAS positional genes associated with at least two CVD-related phenotypes. In bold are genes that showed overlaps in studies where only GWAS studies of cohorts of European ancestry were included. Underlined are genes that showed overlaps under the more stringent GWAS threshold of P<10^−7^.
(DOCX)

Figure S1 Bubble Chart representing the positional GWAS genes intersection in cohorts of European Ancestry only. The size of the phenotype is representative of the percentage of genes studied attributed to that phenotype. Line thickness is representative of the number of intersecting genes between two phenotypes.
(TIF)

Figure S2 Bubble Chart representing the positional GWAS genes Intersection in studies in cohorts of African Ancestry only. The size of the phenotype is representative of the percentage of genes studied attributed to that phenotype. Line thickness is representative of the number of intersecting genes between two phenotypes.
(TIF)

Figure S3 Bubble Chart representing the positional GWAS genes intersection in the ethnicity-pooled analysis with more stringent GWAS P-values<10^−7^. The size of the phenotype is representative of the percentage of genes studied attributed to that phenotype. Line thickness is representative of the number of intersecting genes between two phenotypes.
(TIF)

Figure S4 Bubble Chart representing the GWAS author-reported genes intersection in the ethnicity-pooled analysis. The size of the phenotype is representative of the percentage of genes studied attributed to that phenotype. Line thickness is representative of the number of intersecting genes between two phenotypes.
(TIF)

Figure S5 A plot of 38 genes that overlapped between at least two CVD-related phenotypes based on the PubMed abstracts published up to 2011. Plotted using VIZ-GRAIL. For each plot, phenotypic overlap is arranged along the outer circle; blue indicates three-way or four-way overlaps. Inner circle represents individual genes; genes in bold belong to one of the connecting lines in the middle of circle. The redness and thickness of lines connecting pairs of genes represent the strength of the connections.
(TIF)
