# Supplementary material for: Can Genetic Pleiotropy Replicate Common Clinical Constellations of Cardiovascular Disease and Risk?
Source: PLoS One. 2012 Sep 28;7(9):e46419. doi: 10.1371/journal.pone.0046419 (PMC3460880; doi:10.1371/journal.pone.0046419)
Supplement: Table S1 — Search terms used to select studies from the GWAS catalog by major phenotype. (DOCX) [file pone.0046419.s001.docx]

Supporting Information Table S1: **Search terms used to select studies from the GWAS catalog by major phenotype.**

| **CAD and related traits** | Diastolic blood pressure |
| --- | --- |
| Cardiovascular disease risk factors | Hypertension |
| Coronary artery calcification | Hypertension (young onset) |
| Coronary heart disease | Quantitative traits |
| Myocardial infarction | Tonometry |
| Myocardial infarction (early onset) | Systolic Blood Pressure |
| Major CVD |  |
| Subclinical atherosclerosis traits (other) | **Obesity and related traits** |
|  | Adiposity |
| **CKD and related traits** | Anthropometric traits |
| Biochemical measures | Biomedical quantitative traits |
| Biomedical quantitative traits | Body mass (lean) |
| Chronic kidney disease | Body mass index |
| Chronic kidney disease and serum creatinine levels | Body mass index and fat mass |
| Dialysis-related mortality | Obesity |
| End-stage renal disease | Obesity (early onset extreme) |
| End-stage renal disease (non-diabetic) | Obesity (extreme) |
| Hematological and biochemical traits | Obesity and osteoporosis |
| Nephropathy | Obesity-related traits |
| Other metabolic traits | Quantitative traits |
| Quantitative traits | Waist circumference |
| Renal function and chronic kidney disease | Waist circumference and related phenotypes |
| Select biomarker traits | Waist-hip ratio |
| Serum creatinine | Weight |
| Serum urate |  |
| Serum uric acid | **Type 2 diabetes and related traits** |
|  | Biochemical measures |
| **Hyperlipidemia and related traits** | Biomedical quantitative traits |
| Biochemical measures | Diabetic nephropathy |
| Biomedical quantitative traits | Fasting glucose-related traits |
| Cholesterol | Fasting insulin-related traits |
| Cholesterol, total | Fasting plasma glucose |
| HDL cholesterol | Glycated hemoglobin levels |
| HDL Cholesterol - Triglycerides (HDLC-TG) | Hematological and biochemical traits |
| Hematological and biochemical traits | Insulin response |
| Hypertriglyceridemia | Insulin traits |
| LDL cholesterol | Diabetes (incident) |
| Lipid traits | Diabetes related insulin traits |
| Other metabolic traits | Diabetic nephropathy |
| Quantitative traits | Other metabolic traits |
| Select biomarker traits | Quantitative traits |
| Triglycerides | Select biomarker traits |
|  | Two-hour glucose challenge |
| **Hypertension and related raits** | Type 2 diabetes |
| Biomedical quantitative traits | Type 2 diabetes and 6 quantitative traits |
| Blood Pressure | Type 2 diabetes and other traits |
|  |  |
|  |  |
