# Supplementary material for: Can Genetic Pleiotropy Replicate Common Clinical Constellations of Cardiovascular Disease and Risk?
Source: PLoS One. 2012 Sep 28;7(9):e46419. doi: 10.1371/journal.pone.0046419 (PMC3460880; doi:10.1371/journal.pone.0046419)
Supplement: Table S3 — List of GWAS positional genes associated with at least two CVD-related phenotypes. In bold are genes that showed overlaps in studies where only GWAS studies of cohorts of European ancestry were included. Underlined are genes that showed overlaps under the more stringent GWAS threshold of P<10−7. (DOCX) [file pone.0046419.s003.docx]

Supporting Information Table S3: **List of GWAS positional genes associated with at least two CVD-related phenotypes.**

| **Pheno-1** | **Pheno-2** | **GWAS Positional Genes** | **Gene count for GWAS associations with p<1×10^-5^ (p<1×10^-7^)** | **Hypergeometric (unweighted) p-value** | **Weightedp-value** |
| --- | --- | --- | --- | --- | --- |
| BP | CAD | ***CDH13****,KLHL29****,SH2B3*** | 3 (0) | 0.349 | 0.730 |
| BP | CKD | ***ATXN2****,CUBN,KLHL29,****UMOD*** | 4 (2) | 0.172 | 0.547 |
| BP | T2D | ***MEF2C****,****TGFBR2*** | 2 (0) | 0.783 | 0.962 |
| BP | Lipids | ***ATG4C****,KLHL29* | 2 (0) | 0.797 | 0.964 |
| BP | Obesity | *ATG4C,FOXD3,****MSRA*** | 3 (0) | 0.841 | 0.989 |
| CAD | CKD | *APOB,KLHL29* | 2 (2) | 0.737 | 0.950 |
| CAD | T2D | ***CDKN2BAS,DMRTA1,IRS1,KIAA1486,UBA52P6*** | 5 (2) | 0.247 | 0.682 |
| CAD | Lipids | ***APOB,APOC1,BSND,CELSR2,FAM84B,KLHL29,LDLR,LPL,PCSK9,****PDGFD,****PSRC1,SMARCA4,TRIB1,ZNF259*** | 14 (11) | **<0.001** | **0.002** |
| CAD | Obesity | *DMRTA1* | 1 (0) | 0.996 | 0.999 |
| T2D | CKD | ***C6orf223,GCKR****,RPSAP52,SOX11,****VEGFA*** | 5 (1) | 0.272 | 0.725 |
| T2D | Lipids | ***FADS1,GCKR,NCRNA00262,RPL12P33*** | 4 (4) | 0.700 | 0.957 |
| T2D | Obesity | ***ADAMTS9,BCL11A,C6orf223****,COBLL1,DMRTA1,* ***EIF3FP3,FTO,MAGI1,VEGFA*** | 9 (5) | 0.308 | 0.867 |
| Lipids | CKD | *APOB,****GCKR****,KLHL29* | 3 (3) | 0.707 | 0.948 |
| Obesity | CKD | ***C6orf223,VEGFA*** | 2 (2) | 0.979 | 0.999 |
| Obesity | Lipids | *ATG4C,****TNKS*** | 2 (1) | 0.996 | 0.999 |

In bold are genes that showed overlaps in studies where only GWAS studies of cohorts of European ancestry were included. Underlined are genes that showed overlaps under the more stringent GWAS threshold of P<10^-7^.
