# Supplementary figures and images for: Can Genetic Pleiotropy Replicate Common Clinical Constellations of Cardiovascular Disease and Risk?
Source: PLoS One. 2012 Sep 28;7(9):e46419. doi: 10.1371/journal.pone.0046419 (PMC3460880; doi:10.1371/journal.pone.0046419)

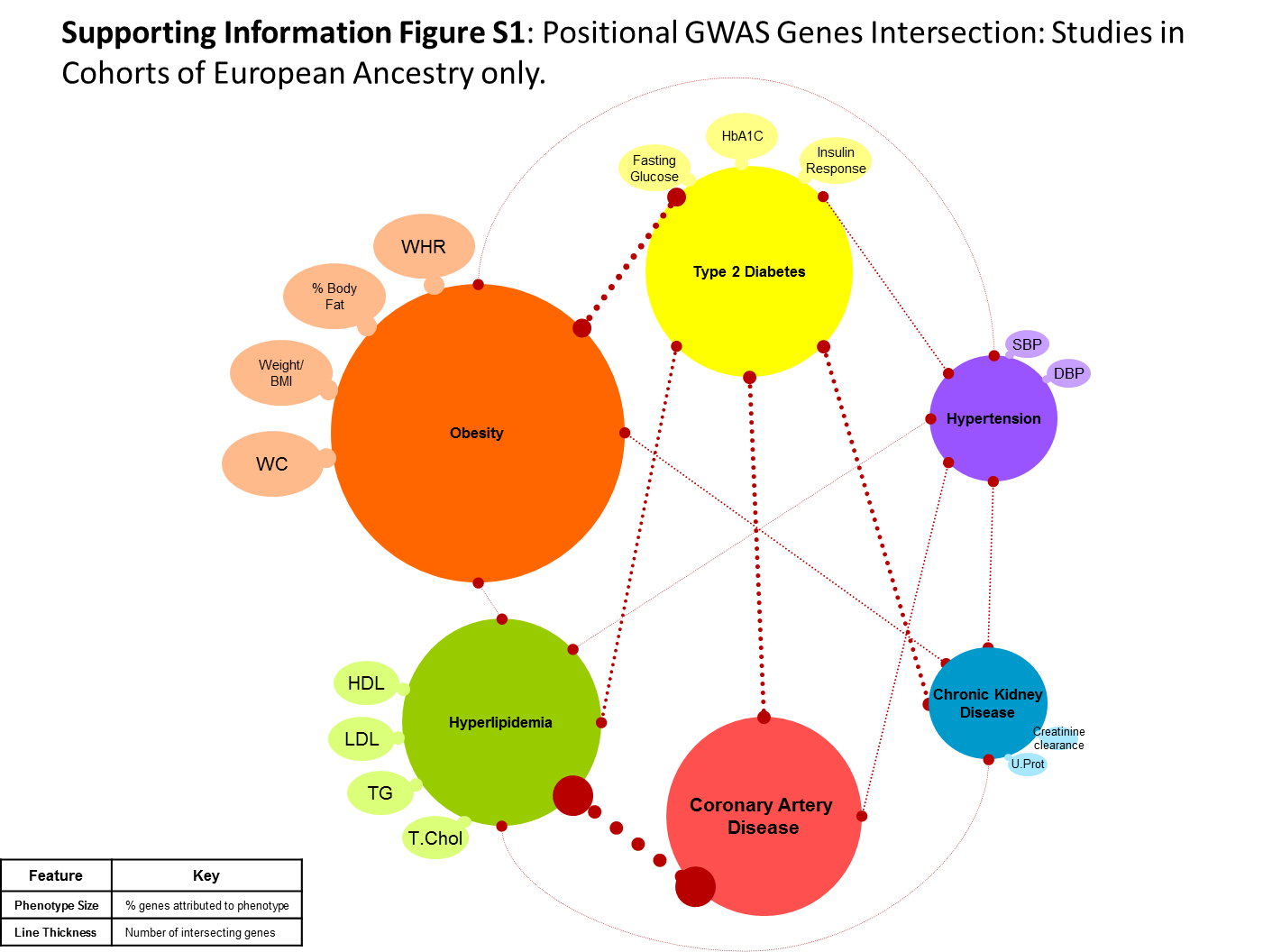

Supplement: Figure S1 — Bubble Chart representing the positional GWAS genes intersection in cohorts of European Ancestry only. The size of the phenotype is representative of the percentage of genes studied attributed to that phenotype. Line thickness is representative of the number of intersecting genes between two phenotypes. (TIF) [file pone.0046419.s004.tif]

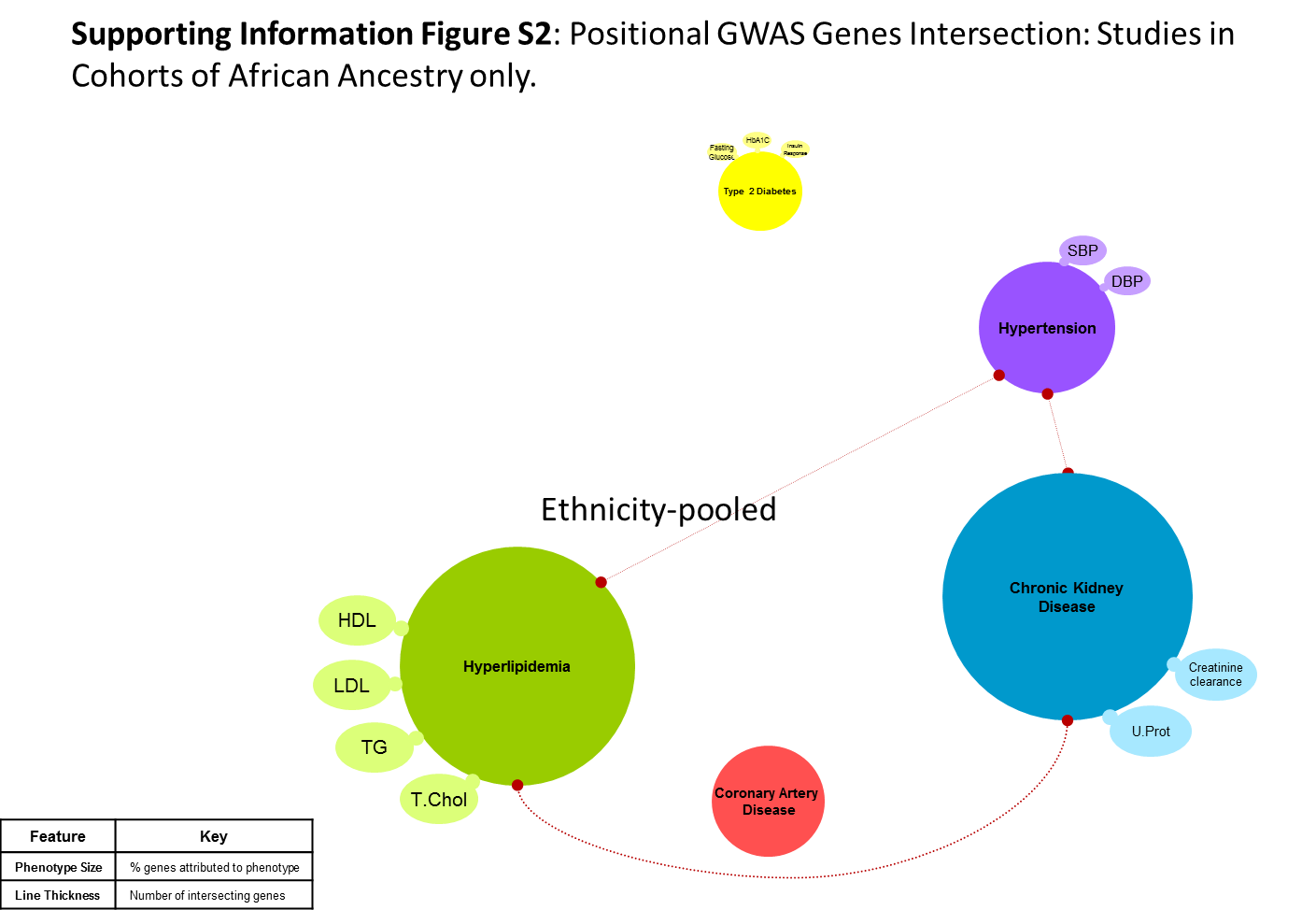

Supplement: Figure S2 — Bubble Chart representing the positional GWAS genes Intersection in studies in cohorts of African Ancestry only. The size of the phenotype is representative of the percentage of genes studied attributed to that phenotype. Line thickness is representative of the number of intersecting genes between two phenotypes. (TIF) [file pone.0046419.s005.tif]

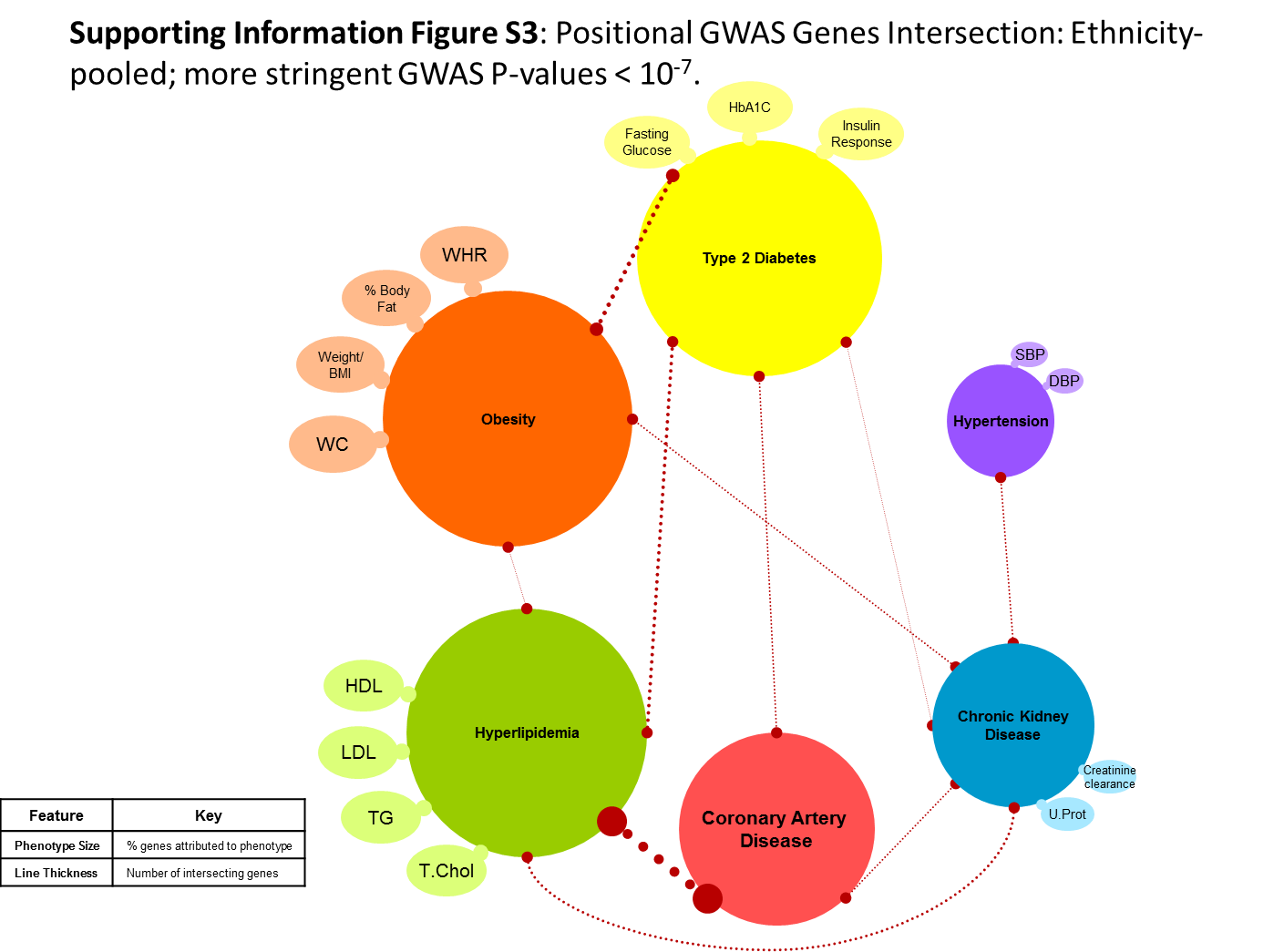

Supplement: Figure S3 — Bubble Chart representing the positional GWAS genes intersection in the ethnicity-pooled analysis with more stringent GWAS P-values<10−7. The size of the phenotype is representative of the percentage of genes studied attributed to that phenotype. Line thickness is representative of the number of intersecting genes between two phenotypes. (TIF) [file pone.0046419.s006.tif]

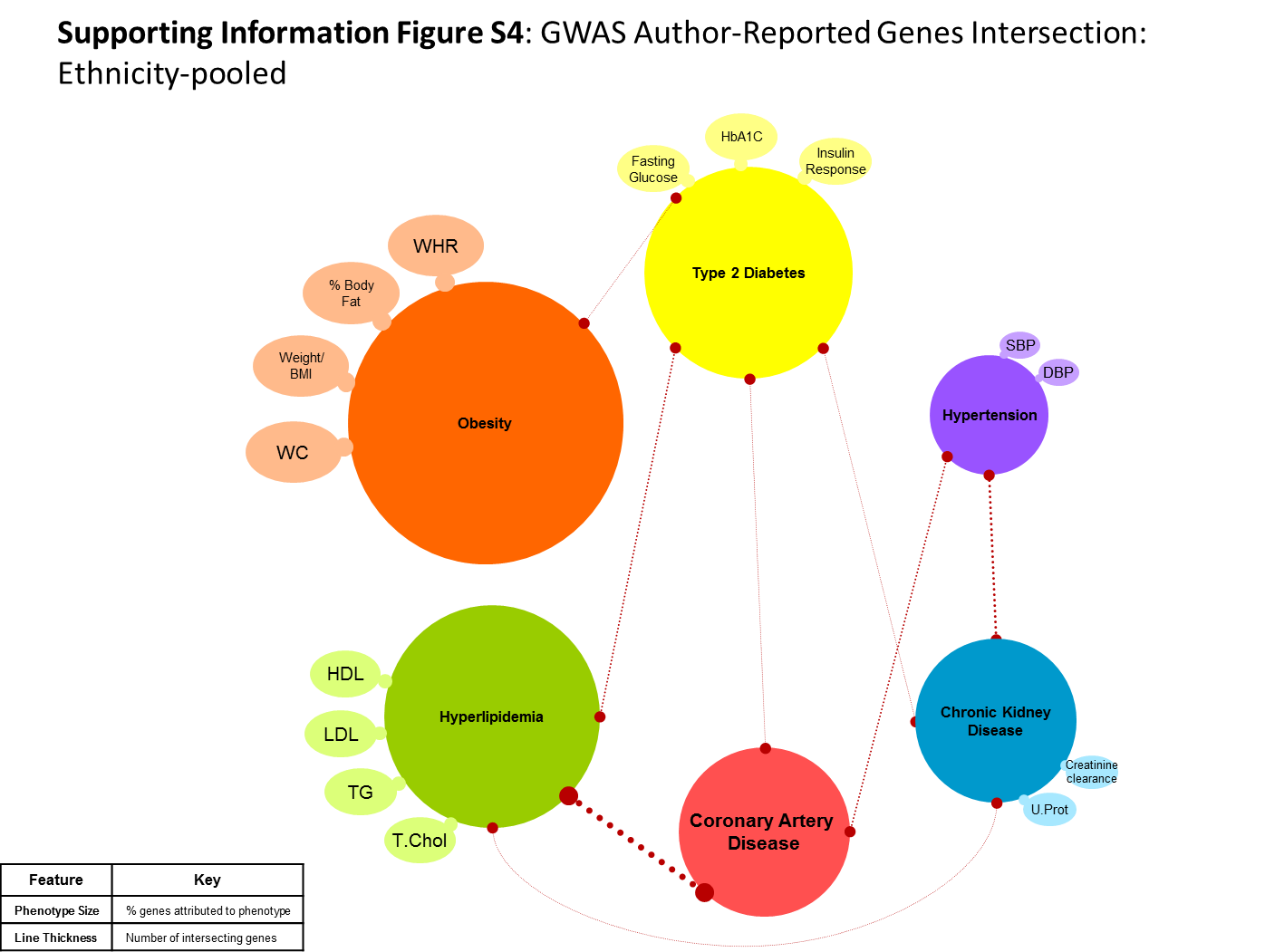

Supplement: Figure S4 — Bubble Chart representing the GWAS author-reported genes intersection in the ethnicity-pooled analysis. The size of the phenotype is representative of the percentage of genes studied attributed to that phenotype. Line thickness is representative of the number of intersecting genes between two phenotypes. (TIF) [file pone.0046419.s007.tif]

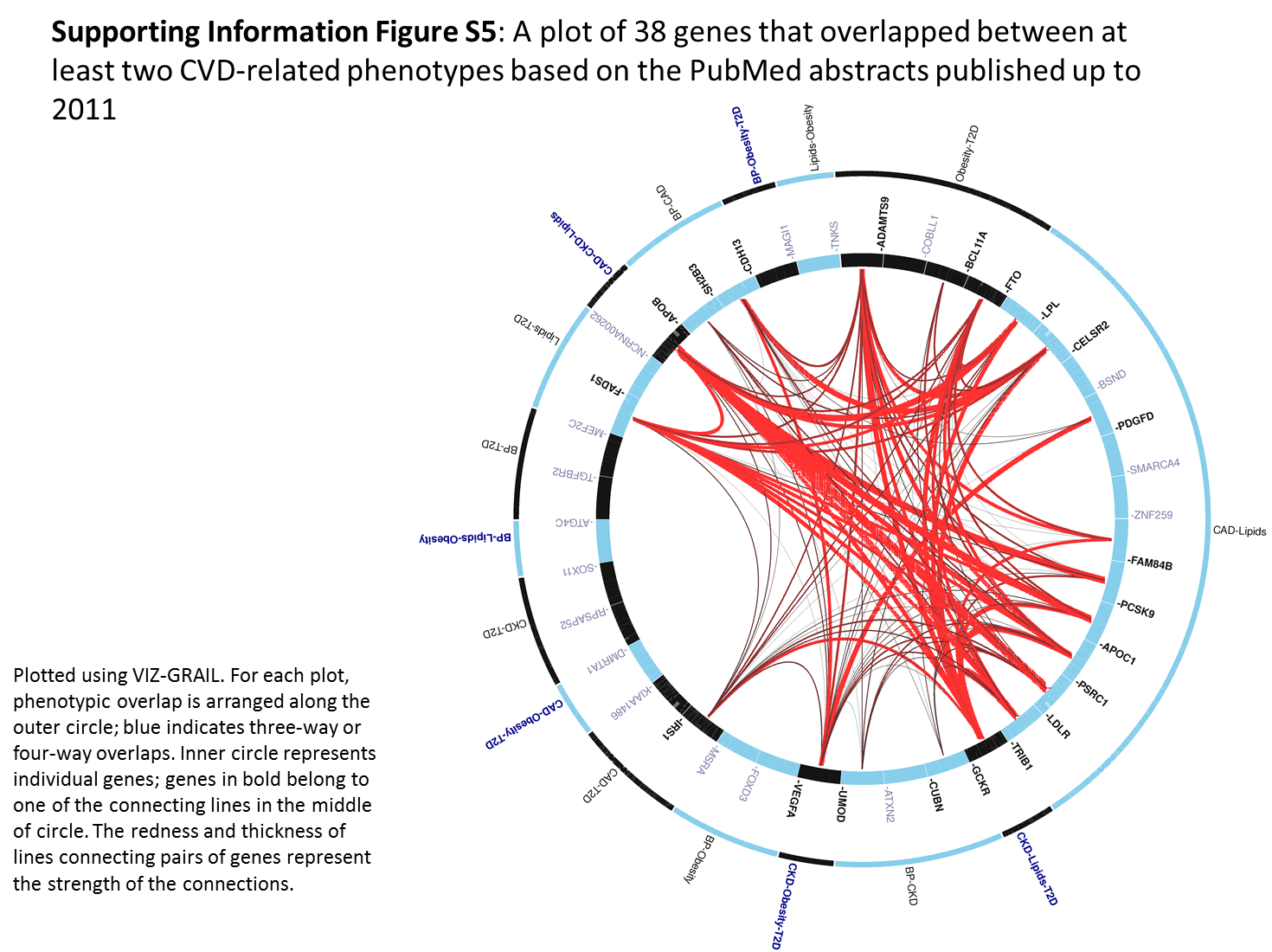

Supplement: Figure S5 — A plot of 38 genes that overlapped between at least two CVD-related phenotypes based on the PubMed abstracts published up to 2011. Plotted using VIZ-GRAIL. For each plot, phenotypic overlap is arranged along the outer circle; blue indicates three-way or four-way overlaps. Inner circle represents individual genes; genes in bold belong to one of the connecting lines in the middle of circle. The redness and thickness of lines connecting pairs of genes represent the strength of the connections. (TIF) [file pone.0046419.s008.tif]
